# Kullback–Leibler Divergence of an Open-Queuing Network of a Cell-Signal-Transduction Cascade

**DOI:** 10.3390/e25020326

**Published:** 2023-02-10

**Authors:** Tatsuaki Tsuruyama

**Affiliations:** 1 Department of Physics, Graduate School of Science, Tohoku University, Sendai 980-8577, Japan; tsuruyam@kuhp.kyoto-u.ac.jp; 2 Department of Drug and Discovery Medicine, Graduate School of Medicine, Kyoto University, Kyoto 606-8507, Japan; 3 Tazuke Kofukai Medical Research Institute, Kitano Hospital, Osaka 530-8480, Japan; 4 Department of Molecular Biosciences, Radiation Effects Research Foundation, Hiroshima 732-0815, Japan

**Keywords:** open queuing network, Kullback–Leibler divergence, signal transduction

## Abstract

Queuing networks (QNs) are essential models in operations research, with applications in cloud computing and healthcare systems. However, few studies have analyzed the cell’s biological signal transduction using QN theory. This study entailed the modeling of signal transduction as an open Jackson’s QN (JQN) to theoretically determine cell signal transduction, under the assumption that the signal mediator queues in the cytoplasm, and the mediator is exchanged from one signaling molecule to another through interactions between the signaling molecules. Each signaling molecule was regarded as a network node in the JQN. The JQN Kullback–Leibler divergence (KLD) was defined using the ratio of the queuing time (λ) to the exchange time (μ), λ/μ. The mitogen-activated protein kinase (MAPK) signal-cascade model was applied, and the KLD rate per signal-transduction-period was shown to be conserved when the KLD was maximized. Our experimental study on MAPK cascade supported this conclusion. This result is similar to the entropy-rate conservation of chemical kinetics and entropy coding reported in our previous studies. Thus, JQN can be used as a novel framework to analyze signal transduction.

## 1. Introduction

A cell-signal-transduction cascade forms a network of reactions that modify protein molecules. The modified protein can diffuse in the cytoplasm and modify another protein. Finally, modified proteins translocate to the nucleus to bind to the promoter in DNA to promote the gene expression encoded in the DNA [1]. Currently, kinetic models based on partial-differential equations (PDEs), including diffusion terms, primarily simulate signal-transduction kinetics and networks. Although PDEs reduce theoretical difficulties through mathematical simplification, the stochastic factors essential to biological kinetics are generally omitted.

The queue in operations is used for analyzing the congestion phenomenon of stochastic systems. For example, queuing theory applies to service-processing design to maintain the waiting time within a specific range [2,3,4,5,6]. The queuing theory applies to a system consisting of a “server”, a “waiting room”, and a “customer” who arrives and stays for a certain time and accepts the “service.” Kendall introduced the symbol of the queue in the form of A/B/C/D (A: the arrival of the customer, B: the distribution of the service time, C: the number of servers, D: the capacity of the system including the waiting room) [7]. Recently, this theory has been applied to analyzing the queuing of packets in routers in communication networks to model gene-expression networks [8]. Furthermore, the theory can apply to the management of the healthcare system [9] in which the patient-waiting time is analyzed when introducing electronic medical-record systems. COVID-19 pandemic infections have been modeled using this theory [10,11]. On the other hand, there have been several studies that have applied queueing networks to biological research [12,13]. For example, the enzymatic reaction [14], molecular birth process [15], metabolism pathway [16], and pharmacokinetics [17] were analyzed using the queueing network. However, few theoretical studies have considered the signal-transduction network and individual chain reactions connected to multiple signaling proteins using QN theory. This study entailed the use of QNs to quantitatively analyze a signal-transduction network. In this regard, the diffusion process of signaling molecules in the cytoplasm and its phosphorylation by other signaling molecules are relevant. The diffusion is the “queue” of signaling molecules formed in the cytoplasm. The phosphorylation process is the service. The network node server is the signaling molecule, and the network service is the process of modifying signaling molecules, which is mediated by the exchange of inorganic phosphate groups between the signaling molecules. Furthermore, the queue-reaction-cascade self-interacts to form a network.

Considering the network of the queue, that is, QN, the theory can describe complex stochastic networks. In particular, this study analyzed a quantitative signal-transduction network for optimizing signal transmission where it was necessary to consider the entire network of queues; therefore, a QN that comprises multiple queues of combined nodes was applied [18,19]. QN is classified into closed and open QN. For closed QN, several customers are exchanged within the QN without arriving or leaving [7]; for open QN, customers receive services from multiple nodes and leave the QN. Jackson’s network (JQN) is an open QN [20]. The theory also applies to the management of cloud computing systems for optimizing networking [2].

This study aimed to create a QN model based on JQN to maximize signal transduction. Previously, I reported that when signal transduction is maximized, the signal-transduction rate is conserved through the cascade based on an entropy-coding system or nonequilibrium thermodynamics and kinetics [21,22].

In this study, we will first review the JQN theory and explain a cellular biological model of signal transduction based on the theory. Second, we will show a theoretical achievement of signal transduction based on the model. Lastly, we will examine the experimental signal-transduction data using the theoretical model. The discussion section will suggest the existence of a relationship between queuing, entropy encoding, and chemical potential.

## 2. Materials and Methods

### 2.1. Cell Culture

We reported the detailed protocol in our previous study [23]. The A431 human skin-cancer cell line was obtained from RIKEN BioResource Research Center (Tsukuba, Japan). A431 cells (0.6 × 105) were cultured in a 5% CO_2_ atmosphere at 37 °C for 5 days. EGF (100 ng/mL; Cell Signaling Technology, Danvers, MA, USA) was added to the cultures and incubated for 0 (untreated), 15, 30, 45, 60, 120, and 180 min. The cell extract was purified using an antibody-array assay kit (Full Moon BioSystems, Inc., Sunnyvale, CA, USA).

### 2.2. Antibody Array Assay

The assay was conducted according to a previously reported protocol [23]. Antibody arrays (PMK185 and PEG214) were used. Biotinylation of the proteins and conjugation and detection by Cy3-streptavidin (PA43001; GE Healthcare Life Science, Little Chalfont, UK) were performed using an antibody-array assay kit (Full Moon BioSystems, Sunnyvale, CA, USA). Cell-extract samples (60 mg) were used. A SureScan Microarray Scanner (G2565CA Microarray Scanner System; Agilent Technologies, Santa Clara, CA, USA) was used for scanning microarrays.

## 3. Results

### 3.1. Queueing Model of Signal Transduction

**Figure 1:**
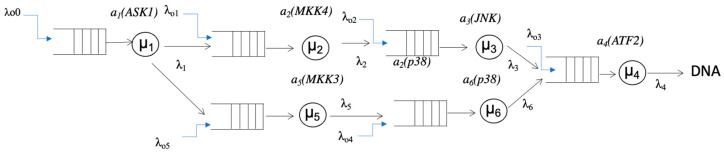
Signal transduction in the JQN model using Kendall’s nomenclature for queuing.

*Cell-signal transduction* is a non-equilibrium process characterized by biological information transmission caused by a biochemical mediator such as adenosine triphosphate (ATP). The signal transduction forms a network of sequential chain reactions that modify signaling molecules, *a_i_*, which constitute the network nodes. Figure 1 illustrates a modeled signal-transduction network. For simplification, the signal is mediated by a mediator *P*. The reaction of signaling molecules, *A_i_,* in the *i*th node (1 ≤ *i* ≤ *n*) is expressed as follows:

(1) $$A_{i}+P\to A_{i}-P:\lambda_{oi}\;$$

(2) $$A_{i}+A_{i-1}-P\to A_{i}-A_{i-1}-P\;:\lambda_{i}\;,$$

(3) $$A_{i}-A_{i-1}-P\to A_{i}-P+A_{i-1}\;:\mu_{i}\;,$$

where *P* diffuses from the outside and arrives at an arrival rate of *λ_oi_* at the *i*th node, *a_i_,* where protein kinase *A_i_* stays (Equation (1)). *λ_i_* is the arrival rate of *A_i_*_−1_-*P* at the *i*th node (Equation (2)), where the signal can be transduced by the exchange of *P* from *A_i_*_−*1*_ to *A_i_* at a rate of *µ_i_* (Equation (3)).

In the actual signaling system, the individual concentrations of the signaling molecules, *A_i_* (1 ≤ *i* ≤ *n*), are sufficiently low; a single signaling event rarely occurs. Therefore, I hypothesized that under the following three assumptions, the signaling event occurs by a Markov process: (i) the probability that a signal event will occur is constant, (ii) the probability that an *i*th step (node) in the cascade occurs at any time interval (*t*, *t* + *Δ*) does not depend on the number of events before time *t* where *Δ* signifies the minimal interval, and (iii) the probability of an event occurring twice or more during a minute’s time *Δ* is negligible and is denoted as *o*(*Δ*). Let the likelihood *p_i_* in the ith step (nodes) in the cascade be the probability that an event occurs *A_i_*-*P* (=*a_i_*) times in time *t* to *t* + *Δ*, and let *λ_i_Δ* be the probability that an event occurs once in a minute, where *Δ* denotes a stable arrival rate. The arrival interval of *p_i_* becomes a random variable sequence that follows the same exponential distribution, and the arrival interval of the signal components is independently and identically distributed:

(4) $$p_{i}\left(t+\Delta \right)=p_{i-1}\left(t\right)\lambda_{i}\Delta +p_{i}\left(t\right)\left(1-\lambda_{i}\Delta \right)$$

Transformation of Equation (4) gives:

(5) $$\;\frac{p_{i}\left(t+\Delta \right)-p_{i}\left(t\right)}{\Delta }\lambda_{i}\left(-p_{i}\left(t\right)+p_{i-1}\left(t\right)\right)$$

Approaching *Δ* → 0 gives a differential equation:

(6) $$\frac{dp_{i}\left(t\right)}{dt}=\lambda_{i}(-p_{i}\left(t\right)+p_{i-1}\left(t\right))$$

Solving Equation (6) gives the solution that *p_i_* is reached by a Poisson distribution:

(7) $$p_{i}\left(t\right)=\prod_{i=1}^{n}\frac{{\left(\lambda_{i}\tau_{i}\right)}^{a_{i}}}{a_{i}!}exp\left(-\lambda_{i}t\right)$$

Equation (7) describes a queuing model in which *A_i-1_-P* arrival rate can be formulated as a Poisson process. Here, the service was defined as the time taken for transcription. In the queue, in accordance with the Poisson process, *λ_i_* is termed as the arrival rate.

The example of JQN in shown (Figure 1). Thus, *P* is transferred by *A_i_* through the network, and the endpoint is the transcription of the nuclear DNA. The diffusion of *P* carried by *A_i_* is the rate-limiting step determining the signal-transduction rate. In this case, *P* is known as a “customer” because it comes from the outside. The exchange rate of *P* from *A_j-1_*-*P* to *A_i_, µ_i_*, is known as the “service rate”. In this way, we assumed that signaling molecules form an open-queue network known as JQN.

Here, the QN of signal transduction satisfies the following conditions of Jackson network: (1) There are phosphorylation “servers” *A_i_*-*P* with the exchange rate *µ_i_* at the *i*th node in the cascade. The arrival rate of *P* at each node *i* is λ*_i_* from the outside of QN according to the Poisson process, and it is possible for *P* to leave the network from any node; (2) there is a customer *P* that is served (transferred to *A_i_*) in the network; (3) *P* in the network is sufficiently high; (4) The service time follows an exponential distribution at all nodes; and (5) The service code is the arrival order of service (FCFS: first-come first-served). In equilibrium state, given that the number of guests arriving per unit time to a node is equal to the number of departures per unit time from that node, we get the following equation, which is called the traffic equation:

(8) $$\lambda_{i}=\lambda_{oi}+\sum_{i=1}^{n}\lambda_{i}p_{i}$$

*λ_i_* (0 *≤ i ≤* 6) represents the arrival rate of *P* from node *a_i_*. *λ_oi_* denotes the arrival rate of *P* from the outside. The ladder-like mark symbolizes the queue of the signaling molecule. *µ_i_* inside the circle represents the exchange (“service” in the queueing theory) rate in which *P* carried by *a_i−_*_1_ is transferred to another signaling molecule *a_i_* at the *i*th node. The arrow represents the orientation of irreversible signal transduction. The final node is DNA (binding histone proteins), where the transcriptional reaction occurs [7]. This is depicted in a figure, and schemes follow the same formatting. The symbol *a_i_* (*A_i_*) above the *µ_i_* represents that signal molecule *A_i_* stays at the node.

Because the signal-molecule arrival rate is sufficiently low, we assumed that the *i*th molecule, *A_i_,* is localized at the *i*th node. Here, we set {***a***} = {*a*_1_, *a*_2_, …. *A_n_*} = {*a_i_*}, which represents the JQN-node state at the steady state and {***a***(*t*)} = {*a*_1_ (*t*), *a*_2_ (*t*), …. *a* (*t*)} = {*a_i_* (*t*)}, which represents the JQN-node state during the signal transduction. 

The vertical axis represents the ratio of the phosphorylated signaling molecule *a_i_* concentration (1 ≤ *i* ≤ 3) to that at the steady state, *a_i_* (*t*)*/a_i_ = ρ_i_* (*t*)*/ρ_i_*, where *a_i_* represents the signaling molecule *A_j_-P* concentration at the steady state. The horizontal axis indicates the duration, where *τ_i_* (1 ≤ *i* ≤ 3) represents the phosphorylation period for each signaling molecule *a_i_.* In this case, the signal transduction proceeds in the order of 1→2→3.

Furthermore, we set *ρ_i_* = *λ_i_/µ_i_*. *ρ_i_* denotes the value at the initiation of the signal event. As mentioned below, *ρ_i_* is equal to *a_i_*. The following expression can be obtained using Jackson’s theorem [20] and the probability that *a_i_* stays at the *i*th node at the steady state:

(9) $$\begin{array}{c} p\left(a\right)=p_{1}\left(a_{1}\right)\;p_{2}\left(a_{2}\right)\cdots \;p_{n}\left(a_{n}\right)\\ =\prod_{i=1}^{n}\;p_{i}\left(a_{i}\right)=\prod_{i=1}^{n}\;{\rho_{i}}^{a_{i}}\left(1-\rho_{i}\right). \end{array}$$

and

(10) $$a_{i}=\frac{\lambda_{i}}{\mu_{i}-\lambda_{i}}=\frac{\rho_{i\;}}{1-\rho_{i\;}}$$

where

(11) $$\rho_{i\;}=\frac{\lambda_{i}}{\mu_{i}}$$

Since the arrival rate is sufficiently low in the entire signal-transduction cascade, *λ_i_/µ_i_* << 1 holds for all *a_i_*. In this case, from Equation (9),

(12) $$\;p_{i}\left(a_{i}\right)≅{\rho_{i}}^{a_{i}}$$

(13) $$a_{i}≅\;\rho_{i\;}$$

and

(14) $$\sum_{i=1}^{n}a_{i}=a$$

The queue length of the *i*th node, *L_i_*, is equal to that of the independent queue. In this case, *L_i_* and the sum of *L_i_*, *L*, are expressed by Little’s formula [24] as follows:

(15) $$L=\sum_{i=1}^{n}\;L_{i}=\sum_{i=1}^{n}\frac{\;\rho_{i}}{1-\rho_{i}}\sim \sum_{i=1}^{n}\;\rho_{i}=a$$

where *λ_i_/µ_i_* << 1, Equations (13) and (14) were used. Likewise, we set the concentration of *a_i_* during the signal transduction:

(16) $$\;\rho_{i}\left(t\right)=\frac{\lambda_{i}\left(t\right)}{\mu_{i}\left(t\right)}=a_{i}\left(t\right)$$

and

(17) $$\sum_{i=1}^{n}\;\rho_{i}\left(t\right)=a$$

**Figure 2:**
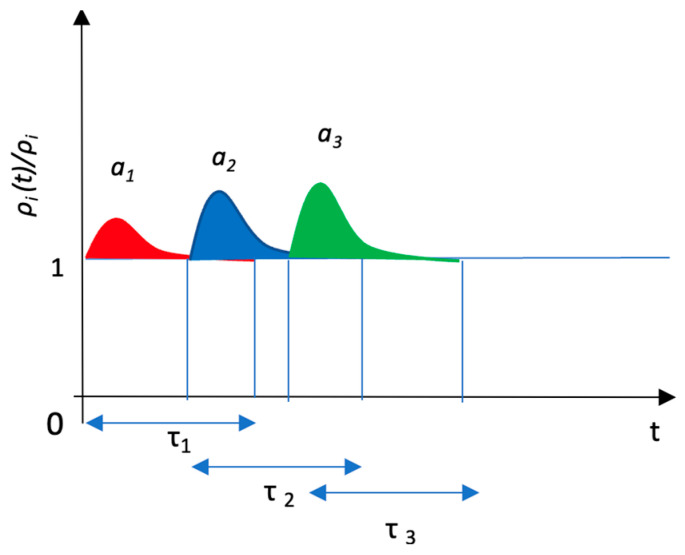
Example of the time courses of signal transduction.

An example of the time courses of signal transduction expressed by *ρ_i_* (*t*)*/ρ_i_* is shown in Figure 2.

### 3.2. Kullback–Leibler Divergence of JQN

Suppose that the signal-network activation in which the *i*th node is activated is obtained. If the complete signal-transduction process is repeated *a* times in the entire signal-transduction system and each signal-transduction process is repeated *a_i_* times in the *i*th node, the probability that the transduction system stays at the given JQN state {***a***} is calculated as follows:

$$\frac{a!}{a_{1}\left(t\right)!a_{2}\left(t\right)!\ldots a_{n}\left(t\right)!}p\left(a\right)=\frac{a!}{a_{1}!a_{2}!\ldots a_{n}!}{\rho_{1}}^{a_{1}}{\rho_{2}}^{a_{2}}\cdots {\rho_{n}}^{a_{n}}$$

Taking the logarithm of the left-hand side, we obtain (Appendix A):

(18) $$\begin{array}{c} \log\left(\frac{a!}{a_{1}\left(t\right)!a_{2}\left(t\right)!\ldots a_{n}\left(t\right)!}{\rho_{1}}^{a_{1}\left(t\right)}{\rho_{2}}^{a_{2}\left(t\right)}\cdots {\rho_{n}}^{a_{n}\left(t\right)}\right)\\ =-a\;\overset{n}{\underset{i=1}{\sum }}\;\rho_{i}\left(t\right)\log\frac{\rho_{i}\left(t\right)}{\rho_{i}}=-aD\left(\rho \left(t\right)||\rho \right) \end{array}$$

Here,

(19) $$D\left(\rho \left(t\right)||\rho \right)\equiv \sum_{i=1}^{n}\;\rho_{i}\left(t\right)\log\frac{\rho_{i}\left(t\right)}{\rho_{i}}\;,$$

which has the form of the KLD. The KLD has been used in sensor and imaging analyses [25], Bayesian model diagnostics [26], clinical trial assessments [27], and information science [28]. We recently performed the EGFR cascade analysis based on the KLD theoretical framework [23]. Furthermore, from Little’s law in queueing theory [24],

(20) $$\tau =\sum_{i=1}^{n}a_{i}\tau_{oi}=\sum_{i=1}^{n}a_{i}\frac{1}{\lambda_{oi}}$$

(21) $$\tau_{io}=\frac{1}{\lambda_{oi}}$$

In Equation (21), $\tau_{oi}$ denotes the duration of a single signal mediator *P* for a single signaling molecule *a_i_*_._ We hypothesized that cells signal more efficiently by eliminating redundancies in terms of energy metabolism. This assumption was also adopted in our previous study [23] and led to maximizing KLD, i.e., information gain, per the signal-event time at each step of the cell-signal-transduction cascade.

To maximize D(ρ(t)||ρ) per a given duration τ, we introduced function *F* with the constraint Equation (20), as given below.

(22) $$\begin{array}{cc} F & =D\left(\rho \left(t\right)||\rho \right)+\overset{n}{\underset{i=1}{\sum }}\;\gamma \;a_{i}\tau_{oi}\\ & =D\left(\rho \left(t\right)||\rho \right)+\sum_{i=1}^{n}\gamma \;{r_{i}}^{a_{i}}\frac{a_{i}}{\lambda_{oi}} \end{array}$$

where γ is an arbitrary parameter independent of node *i.* From Equation (22),

(23) $$\frac{\partial F}{\partial a_{i}}=0$$

Subsequently,

(24) $$\gamma =-\lambda_{oi}\log\;\frac{\rho_{i}\left(t\right)}{\rho_{i}}$$

Using Equation (21),

(25) $$\log\;\frac{\rho_{i}\left(t\right)}{\rho_{i}}=-\gamma \tau_{oi}$$

Accordingly, taking the sum of both sides,

(26) $$D\left(\rho \left(t\right)||\rho \right)=-\sum_{i=1}^{n}\rho_{i}\left(t\right)\tau_{oi}=-\gamma \tau$$

Therefore, we have

(27) $$-\gamma =\frac{D\left(\rho \left(t\right)||\rho \right)}{\tau }$$

The negative value of *γ* indicates the average KLD per signal duration. In conclusion, the average KLD rate per signal duration is independent of the signal step node because *γ* is independent of the step-node number, *i*.

### 3.3. Chemical Potential in JQN

Here, we defined the chemical potential of *a_i_* at the pre-signal event state as

(28) $${\mu_{i}}^{st}≔{\mu_{i}}^{0}+k_{B}T\log\rho_{i}={\mu_{i}}^{0}+k_{B}T\log a_{i}$$

and the signal state as

(29) $$\begin{array}{c} \mu_{i}≔{\mu_{i}}^{0}+k_{B}T\log\rho_{i}\left(t\right)={\mu_{i}}^{0}+k_{B}T\log a_{i}\left(t\right)\;\; \end{array}$$

where *k_B_* denotes the Boltzmann coefficient and *T* represents the temperature of the system. The thermodynamic entropy changes, *Δs_i_,* between the pre-signal and signal event status are given by

(30) $$\Delta s_{i}:=\frac{\mu_{i}-\mu_{i}^{st}}{k_{B}T}=\log\frac{\rho_{i}\left(t\right)}{\rho_{i}}=\;\log\frac{a_{i}\left(t\right)}{a_{i}}$$

Therefore, the entropy changes can be related to KLD [29,30,31]. From Equation (30), we can obtain the relationship between the thermodynamic entropy change and KLD:

(31) $$\Delta S=\sum_{i=1}^{n}\rho_{i}\left(t\right)\Delta s=-\gamma \tau =D\left(\rho \left(t\right)||\rho \right)$$

Accordingly, *γ* has the dimension of a negative entropy-production rate.

### 3.4. Application of KLD Theory to Signal-Transduction Analysis

The mitogen-activated protein kinase (MAPK) signaling cascade (consisting of ASK1, MKK4, JNK, and HSF1) induced by cell stress has been analyzed [32,33,34,35,36,37,38]. The MAPK cascades include the ERK pathway, p38 pathway, and JNK cascade, which are associated with the MAPK stress-response pathway. This pathway is activated by a range of extracellular stress stimuli, including heat shock, oxidation, and exposure to ultraviolet light or radiation [39,40,41]. A MAPK signal cascade in which signal transduction is represented as a sequential activation of the signaling molecules can be described as follows (see Figure 1):

(32) $$\begin{array}{c} ASK1+P↔phospho-ASK1\\ phospho-ASK1+MKK3↔ASK1+Phospho-MKK3\\ phospho-MKK3+p38+ATP↔MKK3+phospho-p38\\ \;phospho-p38+HSF1+ATP↔\;p38+phospho-HSF1 \end{array}$$

where *phospho* represents the phosphorylated status of signaling molecules, and *P* denotes the released phosphate and adenosine diphosphate, respectively. In this cascade, *phosphate* is the mediator. With reference to Equation (32), the average KLD rate of the *ASK*1–*MKK*4–*JNK-HSF*1 cascade is calculated as follows [29]:

(33) $$-\gamma =\frac{\Delta S_{i}}{\tau_{oi}}=\frac{1}{\tau_{oi}}\log\frac{\int_{0}^{\tau_{i}}\rho_{i}\left(t\right)dt}{\int_{0}^{\tau_{i}}\rho_{i}dt}=\frac{1}{\tau_{oi}}\log\frac{\int_{0}^{\tau_{i}}a_{i}\left(t\right)dt}{\int_{0}^{\tau_{i}}a_{i}dt}\;$$

**Figure 3:**
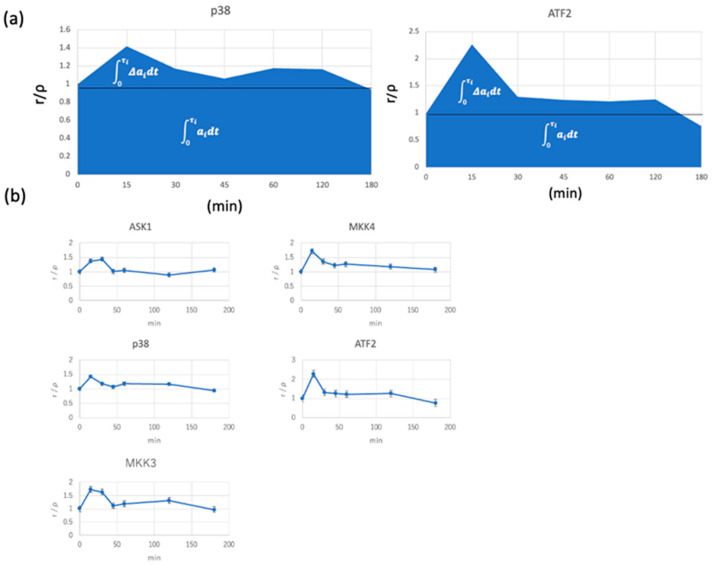
Time course of phosphorylation of signaling molecules.

In the above equation, we used *ρ_i_*(*t*) = *a_i_*(*t*) and $\rho_{i}=a_{i}.$ The integrals in the third term in Equation (33) were calculated using the integral of the plot (Figure 3) (0 ≤ *t* ≤ *τ_i_*). In the experimental study, we cultured the A431 skin-cancer cell line in a serum-free medium and stimulated it with EGFR. In the starved A431 cells, the MAPK-related signal cascade (*ASK*1–*MKK*4–*JNK-HSF*1) was tentatively activated with starvation and minimal EGFR stimuli with EGF. As a result, the KLD rates were around 3.0, which are similar to each other in terms of the Cohen’s factor *d* ≤ 0.5 (Table 1), indicating that Equations (25) and (26) hold in signal transduction.

The vertical axis indicates *ρ_i_*(*t*)/*ρ_i_,* and the horizontal axis shows the time course after the EGF stimulation of the cell. (a) Scheme of the integral of the plot. In terms of the calculation of the integral value, the area above the horizontal line with respect to the vertical axis 1.0 corresponds to the integral value indicated in white, and the lower part corresponds to the integral value indicated in white. The upper end of the integration calculation *τ_j_* was determined by the time of the horizontal axis, and it is estimated that the value of the vertical axis of the plot reaches 1.0. (b) Actual measurement plots of each signaling molecule. The error value indicates the standard error. The measurements were carried out four times.

## 4. Discussion

A primary theoretical approach for analyzing signal transduction in systems biology is the kinetics consisting of continuous differential equations for the time-evolution of the concentration of signaling molecules. Based on some experimental facts, a further numerical simulation was performed by substituting measurable-reaction kinetic coefficients and other parameters. However, in general, the number of signaling molecule proteins in the cytoplasm was small, so concentration fluctuations were expected to be large. Therefore, an analysis using differential equations assuming continuous variables is an appropriate approximation method. In practice, to think of the dynamics of the signaling molecule as a discrete quantity, it is necessary to use the dynamics of discrete parameters, i.e., the stochastic dynamics framework. However, such kinetics are complex and inevitably challenging to apply to complex systems such as cell signaling, which involve non-linear interactions of many signaling molecules, as is the case in systems biology. In contrast, QN theory has a simple framework that can analyze the reaction of discrete quantities. Furthermore, the queues are one-directional, which is consistent with the one-way signaling mechanism in the intracellular biochemical-reaction network, in which the intracellular information is transmitted through the processing of information in the cell membrane or cytoplasm to the nucleus.

If the customer is the signaling molecule itself, a closed QN can be applied where the concentration of the signaling molecule is constant during the signaling period. However, in this case, there are multiple customers, and the QN is more complicated. Besides, each signaling molecule concentration is unstable owing to the fluctuation as aforementioned. To understand the essence of signal transduction and simplify the model, the signal mediators *ATP* or phosphate were treated as customers. Since these molecules are in large quantities in cells and are continuously and constantly supplied to the signal-transduction system from the outside, we applied an open QN. The open QN, where customers arrive externally according to the Poisson process, better reflects the intracellular reaction.

In the application of JQN, when *ρ_i_* < 1 holds for all *ρ_i_* (1 ≤ *i* ≤ *n*), the solution for the probability at the equilibrium distribution is given as a product-form solution. This solution was the basis of this study, shown in Equation (9), and the KLD of the entire system was definable in Equation (19). Since the stationary distribution was in product form and the lengths of the queues of each node at any given time were independent of each other, the process of leaving each node became a Poisson process. It was the first significant development in QN theory, and applying Jackson’s theorem to search for similar product-form solutions in other networks has been the subject of much research. Essentially, a cell-signaling network consists of a considerable number of nodes or phosphorylated (the active form of) signaling molecules, where each service rate (phosphorylation of other signaling molecules) has different values. Thus, JQN represents the signal-transduction system. We applied Little’s formula [2] and Jackson’s theorem [20] to queuing theory. We obtained Equations (26) and (27) in which the JQN KLD per transduction time is constant when the KLD is maximized. As a result, the conclusion was drawn that the KLD rate is constant regardless of the node.

Intracellular signal transduction is a complicated process consisting of a series of modifications and demodification reactions of intracellular proteins. Due to the simplicity of this chain, we introduced the Markov chain (M), i.e., the assumption that the reaction step depends only on the preceding and succeeding steps and that the reaction process formed a model of a queue of M/M/*A_i_*. Furthermore, it was necessary to add thermodynamic information to the model as signal transduction is interpreted as the simultaneous transduction and conversion of biological information. In order to understand the latter, it was necessary to think about fluctuation theorem and entropy coding, and KLD represents the information gain before and after cell stimulation. These concepts are closely related to each other, forming a unified framework of an information theory of intracellular signal transduction. As a result, in this study, an important conclusion was obtained: the KLD per transduction time (code length) in the same cascade signal-transduction step is constant. Importantly, this conclusion was verified by measuring the modification and demodification of proteins before and after cell stimulation.

By introducing quantitative evaluation in this research, it was possible to estimate the transduction time in the signal-conversion step from the fluctuation before and after the whole signal network. Additionally, based on the result that the KLD rate is constant in each step in the known cascade, conversely, we may find a new cascade by comparing the KLD rate of each signaling molecule. For example, when KLD rates of signaling molecules A and B are similar in the given signal event, a signal cascade may be formed between A and B. Further experimental research will be required to validate this idea.

The model of the QN is valid for the following reasons. First, it facilitates the consideration of signal transformations that are irreversible reactions. In this model, the rate-limiting step is a long, reversible process in which a signaling molecule diffuses to the next signaling molecule that is its substrate. The phosphorylation process in service is a short process, which is irreversible in this model. Therefore, the signal never passes through the same node again without considering the feedback mechanism. Such a model framework is useful for signal transduction. Second, protein–protein phosphorylation is a complex non-linear reaction that is difficult to target for kinetic analysis. Therefore, in the application of network theory, we can ignore elementary processes that can be analyzed with simpler models. Third, protein molecules have large concentration fluctuations and are not subject to simple kinetics. This framework overcomes these problems and enables a unified analysis.

We focused on a stress-induced *ASK*1–*MKK*4–*JNK-HSF*1 signal-transduction cascade and found that the average information gain, i.e., KLD, per signal duration is consistent in the pathway, indicating the conservation of KLD rate. Conversely, the cell signal-transduction system proceeds in circumstances in which the rate of signal transduction is maximized. By observing the temporal variation of the phosphorylation of individual signaling molecules, plotting the increase against time, and taking the logarithm of the integral value, the KLD increase rate per hour can be comprehensively measured. As a result, signals can be transmitted between signaling molecules with similar KLD growth rates. This is one of the methods that makes it possible to discover unknown cascades between so many signaling molecules. Furthermore, by changing the dose of radiation that produces reactive oxygen, it is possible to identify signaling molecules that show an increase in the KLD rate correlated with the radiation dose and to more accurately identify cascades specific to the type of stress.

Previously, we applied entropy encoding to a code-string model of the signal cascade and found that the entropy-production rate is conserved [42], which corresponds with the conclusion of this study where the KLD rate for the queue is consistent regardless of the node, *a_i_.* Also, we reported that the non-equilibrium kinetics of signal transduction indicates that the entropy-production rate is consistent in the signal-transduction cascade [22,23]. According to Equations (30) and (31), KLD can be expressed by a chemical potential change and thermodynamic entropy *ΔS* in the entire signal transduction. In summary, the entropy-production rate conservation holds in the JQN, entropy-coding theory, and non-equilibrium thermodynamics. This indicates an intriguing relationship between queuing, entropy encoding, and chemical potential.

A limitation of the present study is that we used continuous derivative operations in deriving Equations (26) and (27) rather than discrete operations; therefore, this mathematical evaluation should be further verified. A detailed study, replacing the derivative operation with a discrete operation, may be explored in future work.

In conclusion, the JQN can be used for a quantitative analysis of signal transduction.

## Figures and Tables

**Table 1:** Fundamental statistics of KLD rates.

	Mean (μ)	Standard Deviation
ASK1 (Phospho-Ser83)	3.27	0.76
MKK4 (Phospho-Thr261)	3.43	0.70
p38 (Phospho-Thr180)	3.07	0.73
MKK3(Phospho-Ser189)	3.19	0.20
ATF2(Phospho-Thr69 or 51)	3.03	0.50
Negative control	0.00	0.00
	**Δμ**	**Cohen’s Factor d**
ASK1 (Phospho-Ser83)-MKK4 (Phospho-Th261)	0.16	0.22
MKK4 (Phospho-Th261)-p38 (Phospho-Thr183)	0.36	0.50
MKK3(Phospho-Ser189)-p38	0.12	0.22
p38 (Phospho-Thr183)-ATF2(Phospho-Thr69 or 51)	0.04	0.06

## Data Availability

Suggested Data Availability Statements are available in section Supplementary Materials.

## Supplementary Materials

The following supporting information can be downloaded at: https://www.mdpi.com/article/10.3390/e25020326/s1, Figure S1: The vertical axis indicates *ρi(t)/ρi*, and the horizontal axis shows the time course after the EGF stimulation of the A431 cell. The error value indicates the standard error. The measurements were carried out four times. The mean of KLD rate in (a), (b), (c), and (d) were 3.52, 3.06, 2.79, and 3.43.

## Appendix A. Calculation for Equation (18) in the Text

The following is the calculation deriving for Equation (18).

$$a!=a^{a}\exp\left(-a\right)\sqrt{\left(2\pi a\right)}\left(1+O\left(1/a\right)\right),$$

$$a_{i}\left(t\right)!=a_{i}{\left(t\right)}^{a_{i}\left(t\right)}\exp\left(-a_{i}\right)\sqrt{\left(2\pi a_{i}\right)}\left(1+O\left(1/a_{i}\right)\right),$$

Accordingly,

$$\begin{array}{c} \log\frac{a!}{a_{1}\left(t\right)!a_{2}\left(t\right)!\ldots a_{n}\left(t\right)!}{\rho_{1}}^{a_{1}\left(t\right)}{\rho_{2}}^{a_{2}\left(t\right)}\cdots {\rho_{n}}^{a_{n}\left(t\right)}\\ ≅\log\prod_{i=1}^{n}{\left(\frac{\rho_{i}\left(t\right)}{\rho_{i}}\right)}^{-a_{i}\left(t\right)}\frac{1+O\left(\frac{1}{a}\right)}{\sqrt{{\left(2\pi a\right)}^{n-1}\prod_{i=1}^{n}\rho_{i}\left(t\right)}}\\ ≅\log\prod_{i=1}^{n}{\left(\frac{\rho_{i}\left(t\right)}{\rho_{i}}\right)}^{-\rho_{i}\left(t\right)}\\ =\overset{n}{\underset{i=1}{\sum }}\log{\left(\frac{\rho_{i}\left(t\right)}{\rho_{i}}\right)}^{-\rho_{i}\left(t\right)} \end{array}$$

In the above, we used Equations (16) and (17).
