# Supplementary material for: Kullback–Leibler Divergence of an Open-Queuing Network of a Cell-Signal-Transduction Cascade
Source: Entropy (Basel). 2023 Feb 10;25(2):326. doi: 10.3390/e25020326 (PMC9955153; doi:10.3390/e25020326)
Supplement: Supplementary file 1 [file entropy-25-00326-s001.zip › entropy-2178528-supplementary.pdf]

data

MDPI

Data Descriptor

Kullback–Leibler Divergence of an Open-Queuing Network of a Cell-Signal-Transduction Cascade

Tatsuaki Tsuruyama [1,2,3,4]

1 Department of Physics, Graduate School of Science, Tohoku University, Sendai 980-8577, Japan
2 Department of Drug and Discovery Medicine, Graduate School of Medicine, Kyoto University, Kyoto 606-8507, Japan
3 Tazuke Kofukai Medical Research Institute, Kitano Hospital, Osaka 530-8480, Japan
4 Department of Molecular Biosciences, Radiation Effects Research Foundation, Hiroshima 732-0815, Japan

Abstract: The additional data that should have been shown in the article is provided.

1. Summary

This supplementary figure 1 presents the additional phosphorylation data for signaling molecules of the ASK1–MKK4–JNK-HSF1 cascade. There are no additional data on p38 kinase and MKK3 phosphorylation other than the data presented in the text. EGFR stimulation experiments under the same conditions showed values similar to those shown in the text, regardless of the type of phosphorylated amino acid residue. The additional supported the conclusion of KLD rate conservation.

2. Data Description

2.1. Time course of phosphorylation of signaling molecules

The vertical axis indicates $\varrho_i(t)/\varrho_i$, and the horizontal axis shows the time course (min) after the EGF stimulation of the cell. The error value indicates the standard error. The measurements were carried out four times. The mean of KLD rates in (a), (b), (c), and (d) were 3.52, 3.06, 2.79, and 3.43.

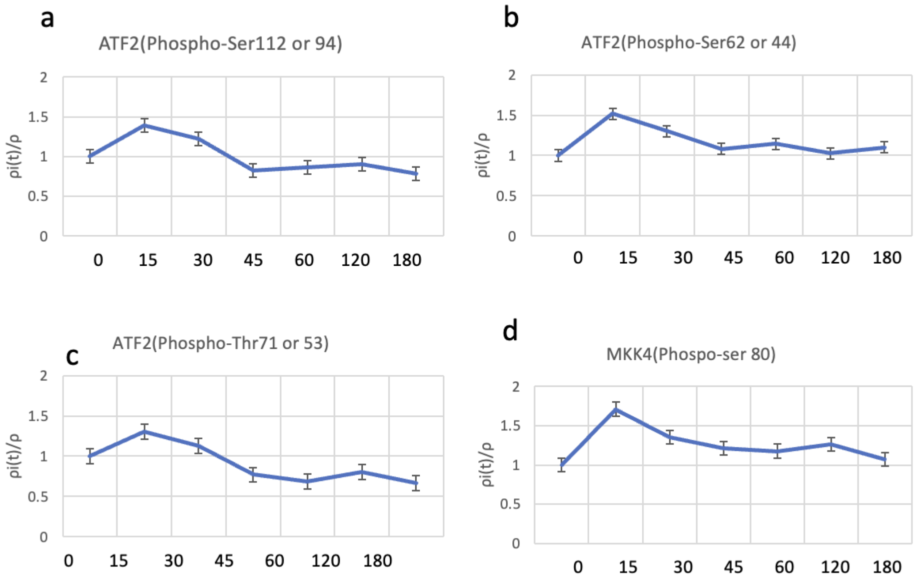

Supplementary Figure S1. The vertical axis indicates $\rho i(t)/\rho i$, and the horizontal axis shows the time course after the EGF stimulation of the A431 cell. The error value indicates the standard error. The measurements were carried out four times. The mean of KLD rate in (a), (b), (c), and (d) were 3.52, 3.06, 2.79, and 3.43.

Funding: This research was funded by a Grant-in-Aid from the Ministry of Education, Culture, Sports, Science, and Technology of Japan (Synergy of Fluctuation and Structure: Quest for Universal Laws in Non-Equilibrium Systems, P2013-201 Grant-in-Aid for Scientific Research on Innovative Areas, MEXT, Japan). This study was supported by the Kitano Medical Institute and the Radiation Effects Research Foundation (RERF), Hiroshima and Nagasaki, Japan, a public interest foundation funded by the Japanese Ministry of Health, Labour, and Welfare (MHLW) and the US Department of Energy (DOE). The views of the author do not necessarily reflect those of the two governments.

Institutional Review Board Statement: Not applicable" for studies not involving humans or animals.

Informed Consent Statement: Not applicable" for studies not involving humans.

Conflicts of Interest: The funders had no role in the design of the study; in the collection, analyses, or interpretation of data; in the writing of the manuscript; or in the decision to publish the results".

References

1. Kiso-Farnè, K.; Tsuruyama, T. Epidermal growth factor receptor cascade prioritizes the maximization of signal transduction. *Sci. Rep.* **2022**, *12*, 16950.
